# The COVID-19 Pandemic: A Month of Bioethics in Finland

**DOI:** 10.1017/S0963180120000432

**Published:** 2020-04-30

**Authors:** MATTI HÄYRY

## Abstract

Finland’s first COVID-19 infection was recorded in late January 2020. The person infected was a tourist from China in Lapland. Authorities recommended regular handwashing, coughing in one’s sleeve, not touching your face, physical distancing, and home lockdown for those at risk. The pandemic spread at different paces in different regions, and the first Finnish fatality was recorded on March 20 in Uusimaa province, where the number of documented infections was considerably higher than elsewhere. In late March, the parliament granted the government emergency powers for swift regulations and restrictions. Uusimaa province was isolated from the rest of the country for a fortnight, restaurants were closed, meetings of more than 10 people were forbidden, and schools and universities assumed distant-working modes, as did businesses and civil services where this was feasible.

Finland’s first COVID-19 infection was recorded in late January 2020.^1^ ^,^^2^ The person infected was a tourist from China in Lapland. Authorities recommended regular handwashing, coughing in one’s sleeve, not touching your face, physical distancing, and home lockdown for those at risk. The pandemic spread at different paces in different regions, and the first Finnish fatality was recorded on March 20 in Uusimaa province, where the number of documented infections was considerably higher than elsewhere. In late March, the parliament granted the government emergency powers for swift regulations and restrictions. Uusimaa province was isolated from the rest of the country for a fortnight, restaurants were closed, meetings of more than 10 people were forbidden, and schools and universities assumed distant-working modes, as did businesses and civil services where this was feasible.

The government’s decisions were, according to their own announcements, guided by public health knowledge provided by the Finnish Institute for Health and Welfare (THL). THL is a publicly funded “expert agency that provides … information on health and welfare for decision-making and activities in the field.”^3^ It seems that at least well into April the intention was to slow down the spread of the pandemic so that intensive-care units in hospitals would not be overloaded and thus as many lives as possible could be saved. Meanwhile in Sweden (Finland’s neighboring country), authorities had kept interferences to a minimum with the apparent aim to develop a sufficient herd immunity. Although fewer restrictions were known to increase the death toll among vulnerable groups, the Finnish government started toward the end of my observation period reconsidering some of them.

Finnish people accepted, by and large, the shutdown of normal life and activities without too much resistance. Parents of smaller children did not want to spend the days at home with their offspring and complained about the closure of schools and daycare centers. Some Uusimaa residents who wanted to visit their cottages outside the province expressed their unhappiness. Otherwise, more animated reactions focused on incompetence and unclarities in the public purchase of face masks, and the distribution of public emergency funding to consulting businesses and other ostensibly undeserving recipients.

Apart from protests against inconvenience and ineptitude, concerns about income, livelihood, and the future of businesses began to emerge early on. In the big picture, national and global economies will bounce back sooner or later, if nothing truly unexpected occurs. Private lives and smaller businesses, however, may suffer irredeemable setbacks. Recessions leave long trails of misery, which usually concentrate on groups that are already vulnerable. Partly fueled by this, political systems may be threatened. If liberal democracies cannot handle the aftermath of the pandemic to everyone’s satisfaction, perceived elites may come under increased pressure, rule of law may be challenged, and inclusiveness and participatory governance may become unfashionable, with unpredictable consequences. Whether this is good or bad remains to be seen.

## Saturation for Bioethical Firefighting

The run-of-the-mill bioethical discussion on the COVID-19 pandemic in Finland quickly reached its saturation point. Utilitarian and rights-based solutions were paraded, civic duties evoked, and the virtue of obedience stressed. In popular contributions by academic philosophers and jurisprudents, colleagues have risen up to the challenge and turned into the firefighters that Tuija Takala predicted in her 2005 article “Demagogues, firefighters, and window dressers: Who are we and what should we be?”^4^ Takala’s idea was that bioethics as an academic enterprise has been dead or dormant for decades and that the so-called bioethicists among us either preach their own ideology, run from crisis to crisis verbalizing popular concerns in their own pet language, or contribute to drawing attention away from wider economic and political issues by digging up and addressing solvable local concerns in medicine and healthcare. Firefighting, the middle approach, is a solution for exceptional times, and that is what Finnish bioethicists have chosen to do for now.

## Standard Ethical Theories and the Isolation of a Province

All standard ethical theories made cameo appearances in the discussion,^5^ although they were not exhaustively analyzed. Let us see what their verdicts on the Finnish government’s isolation of Uusimaa province could have been.

*Act utilitarianism* demands that all our individual choices must aim at maximizing measurable good. By setting up the restrictions (*prima facie* bad), the government tried to slow down the spread of the pandemic (good?), promote public health (good), save lives (good), maybe at the expense of losing other lives (bad). Whether applying the brakes at the time when it was done will turn out to be good or bad can be reliably assessed only when the pandemic is over. Empirical calculations might retroactively support the isolation, but the possible sacrifice of some innocent lives will still leave some moral residue.

*Rule utilitarianism* instructs us to device principles on which to act in particular cases. One such principle could be the use of Quality Adjusted Life Years (QALYs) in decisionmaking. If those who die are very old, they will not dent the national QALY aggregate as much as the young and healthy. Sweden seems to be following this policy. Another option would be to devise some kind of utilitarianism with side constraints. Basic human rights, for instance, could set the limits of seeking health and economic utility. Due to international treaties, both Finland and Sweden swear by this rule, but lawyers and philosophers have questioned whether it has been applied during the pandemic.

*Moral legalism* is not really an ethical theory, but it informs citizens’ thinking in some parts of the world, including Finland. According to it, we only need to obey prevailing laws to be moral. This is compatible with the jurisprudential creed of legal positivism, which states that the law is the law because it is the law, and no further justification is possible or desirable. The idea is not, on the other hand, compatible with natural law theory, which teaches that morality is above existing law and that some laws are bad and need not or ought not to be followed. Human rights lawyers criticized the Finnish government’s restrictions by arguing that they were in defiance of international human rights legislation. Some philosophers objected and appealed to higher moral grounds.

*Kantian ethics* requires us to respect humanity in ourselves and in others. We must act so that we never use humanity as a mere means, but always also as an end in itself. Did the government abide by this rule? Both sides were quickly argued. Yes, because the possible loss of life in the isolated province was not the cause of health benefits elsewhere. No, because the vulnerable were put in harm’s way to benefit others. The magic—and challenge—of the Kantian formula is in the expressions “mere means” and “also as an end,” which can be interpreted in more ways than one.

*Natural law ethics* forbids us to violate or disregard our basic human goods, which are, in the traditional formulation, survival, health, shelter, having and raising offspring, and seeking knowledge, especially concerning God. Insofar as the government’s decision was about protecting lives and health, this approach readily supports them. Since the same family of theories has also produced the doctrine of double effect for exceptions, however, it is not always clear how the principles should be applied.

*Virtue ethics* in its Aristotelian formulation advises us to seek a Golden Mean between the extremes of doing too much or feeling too strongly and doing too little or feeling too weakly. This idea works relatively well in, say, threatening situations: we should have a proper amount of courage instead of being rash (too much action, too little fear) or timid (too little action, too much fear). It does not, however, provide an exact guide for political choices. A care–ethical interpretation might be more promising. This insists that we should identify and recognize vulnerable groups and cherish special relationships. Taking these into account in public as well as in private decisionmaking would prompt us to protect groups that are in particular danger. Since the elderly and people with disabilities are in particular danger, care ethicists could join forces with the human rights lawyers in criticizing the isolation of Uusimaa province.^6^ ^,^^7^

All these views were briefly paraded in the Finnish discussion, but somehow, inexorably, the most visible debate kept revolving around utilitarian and related themes.

## The Utilitarian Case and its Critics

Up until late April, the Finnish government followed a script written predominantly by THL. THL is, by definition, a health utilitarian agency. As a default value, it aims at minimizing morbidity and mortality among the population and maximizing health and wellbeing. It may occasionally recognize the plight of minorities or vulnerable populations, but in a crisis like the COVID-19 pandemic, it prioritizes lives, health, and (time and resources permitting) QALYs. The government and its advisers should aim impartially at the good of as many citizens as possible, and survival and health are the priority.^8^

Utilitarian approaches like this are open to criticism from other ethical and legal points of view, particularly from the angle of basic rights. At the time of the government’s decision to isolate Uusimaa province, two lawyers, Martin Scheinin and Pauli Rautiainen, argued that human rights were under threat.^9^ The public justification of the isolation was to temporarily confine the pandemic, to give hospitals more time to adjust to the situation, and to reduce the risk of overcrowding intensive-care units. Scheinin and Rautiainen pointed out, however, that this decision ignored the human rights of some people in recognized risk groups, especially the elderly. Some of them could have coped better with access to their summer cottages outside Uusimaa. Restricted to their city environments, they could have caught the disease and even died. In the latter case, the government was, to use the ethical metaphors that the lawyers employed, playing God and turning the trolley to sacrifice the few in order to save the many. Scheinin and Rautiainen also stated, among other things, that if even one person dies due to a government decision, it ought not to be made.

Later on, the discussion on the plight of the elderly evolved beyond the few who could have benefited by freedom of travel. The quality of life in nursing homes without visitors, proxy advance directives by telephone conversations with the relatives, the number of COVID-19 deaths among the very frail, and the healthcare system’s policy of not sending them to hospitals for intensive care all raised concerns.

## The Semi-Utilitarian Comeback—Kill if You Do, Kill if You Do Not

Back to the early days of the isolation of Uusimaa, however. Philosopher Antti Kauppinen refused to accept Scheinin and Rautiainen’s view and formulated, first by himself,^10^ then with his colleague Simo Kyllönen,^11^ and then again on his own^12^ objections that rested on greater benefits but, according to their authors, were not blatantly utilitarian. Kauppinen began by criticizing the lawyers’ use of the trolley example attributed to Philippa Foot.^13^ A runaway trolley threatens to kill five people on the track. At the switch of a lever, a bystander can turn the trolley on a sidetrack and save those on the main track. Unfortunately, a person on the sidetrack will be killed. The rough-and-ready utilitarian solution to sacrifice the one seems coldhearted. Letting the five die seems unintuitive. Kauppinen pointed out that Foot was not rejecting the choice to save five lives at the expense of one, but that she was rather trying to show how this can be condoned without drifting into wholehearted consequentialism and utilitarianism.

The theoretical device Foot employed to make her point was the doctrine of double effect.^14^ According to this, an act that has two outcomes, a good one and a bad one, is permissible if and only if (1) it is in and by itself morally good or at least neutral; (2) the bad outcome is not directly willed or intended; (3) the good outcome is not a consequence of the bad outcome; and (4) the good outcome is proportional to the bad outcome.

Both the two lawyers and Kauppinen initially focused on the deaths caused by government action and inaction as the good and bad outcomes. If we accept this limitation, we can analyze the four points as follows (in reverse order).^15^

4) If the Finnish government’s decision to isolate Uusimaa province was based on a well-founded belief that considerably more human lives could be saved by the restriction, the condition seems to be fulfilled. Was the decision based on such a belief? We may never know—governments act on many grounds. Was the belief well-founded? This is not self-evident. But if it was, clear sailing so far.
3) If the government aimed at saving many lives at the expense of a few in Uusimaa, the condition is fulfilled. The deaths of those in Uusimaa do not in any way cause the survival of others. To compare, the doctrine does not permit torture to find out where the next lethal terrorist bomb is. The bad (torture) would in this case be a causal factor to the good (the prevention of the attack). The isolation (as far as lives are concerned) does not follow this logic.
2) The government did not will or intend the deaths of the few in the sense specified by the doctrine. The measure is how the decisionmaker responds to the bad outcome not happening. Would it be a drawback? Would further action be undertaken to make it happen? In the isolation case, the government would have been quite content with zero deaths. Again, the condition is fulfilled, if we define the bad effect exclusively in terms of lives.
1) Is the act in and by itself morally good or at least neutral? Now this is interesting. Someone in the ensuing conversation noted that causing deaths can never be a good thing, whether by act or by omission.^16^ Point taken, but within the doctrine of double effect we are not allowed to focus on consequences at this point. We have to determine the morality of the choice by other means. Foot’s idea apparently was that switching the lever is neutral. The Finnish government’s decision could be a different matter, though. Setting up roadblocks and restricting citizens’ movement, forcibly if needed, does not sound all that innocuous.

## The Semi-Utilitarian Comeback Defeated—Other Values?

The Finnish bioethical discussion on COVID-19 was at its early stages dominated by death and dying. When, however, we look more closely at what the government (1) “did” and (2) “willed and intended,” we can partly advance past counting lives.

Although loss of life was not a part of what the government did and willed by isolating Uusimaa, restrictions of movement are. The aims of the operation could not, according to the government’s counsellors, have been reached without ordering people to stay in their own region and ceasing them if they attempted to cross the border. Talking in terms of the doctrine of double effect, this challenges conditions (1) and (2). Since the deed is curtailing civic liberty by employing the police and the military *and* since the curtailment must be willed and intended, the doctrine does not apply.

## Good, Greater Good, Greatest Good

After Kauppinen’s double effect view had been questioned, he and coauthor Kyllönen formulated a new justification. They argued, in Finland’s most widely distributed newspaper, that the isolation decision was correct because

in moral philosophy, it is widely acceptable to apply the so-called principle of the smallest harm: if, to save a larger group, we have to do something that is not as such wrong, this is permissible even if, as a side effect, someone would come to harm.^17^

This is an interesting statement in many respects (not least because “the principle of the smallest harm” gets less than a dozen hits in an internet search, none of them linked with moral philosophy).

Let us start with the “not as such wrong” caveat, which is evoked here although the authors had been made aware of objections to this.^18^ In liberal creeds, restrictions of freedom are seen as *prima facie* wrong, that is, wrong until proven otherwise, or wrong unless a sufficiently strong justification is given. The fact that the “as such” caveat still persists in the argument has two interpretations. The authors may have had a different “deed” in mind. Perhaps they thought that the government simply “issued an administrative order,” and that issuing administrative orders is, as such, morally good or neutral. Even apart from the doctrine of double effect, however, this is a strained reading. The decision was meant to keep people on two sides of the border, and it was a deliberate curtailment of liberty. The other interpretation is that Kauppinen and Kyllönen do not subscribe to the view that restrictions of freedom are *prima facie* wrong. In that case, their view reduces to simple aggregative utilitarianism, an understanding they support by writing before and after the cited passage:

The aim of the isolation decision is … to protect the life and health of as many Finns as possible. As a side effect, someone in Uusimaa may die – someone who would otherwise have survived. [But due to the principle of the smallest harm] we do not violate [their] right to life, because we are not intentionally taking lives, but protecting lives in a way that unfortunately results in the death of others.^19^

The “not intentionally” here may be a little disingenuous. The government did not mean to kill people in the vulnerable groups, but does this make a moral difference if their demise was a well-known and preventable consequence? We are back at the trolley lever, and Kauppinen and Kyllönen want to switch it to kill the one on the sidetrack. Philosophically speaking, they are welcome to do so, but the justification they present is utilitarian, and the question arises, “In which other circumstances would it be morally right to sacrifice a minority to benefit the majority?” This is the question over which aggregative utilitarianism has stumbled time and again.

## A Note on the Complexities of Utilitarianism

In late April 2020, the discussion, in anticipation of government lifts on restrictions, was moving toward defining the good beyond immediate health concerns. What ends should we aim at? When? Which outcomes should we avoid? Although the Swedish way of building up herd immunity by “sacrificing the elderly” was frowned upon, THL started suggesting that more healthy people also in Finland ought to be allowed to be infected.^20^ The logic was that another attack of the pandemic in a few months’ time could be prevented by doing so. Economists argued that businesses should be allowed to recover in less constrained circumstances. Educators began to worry that children’s learning results suffer with the schools closed. Psychologists warned about the detrimental effects of distance working and home schooling. In the face of these challenges, Kauppinen, true to form, published another opinion piece, this time in an esteemed weekly magazine, insisting that the prevailing restrictions were the right ones and should only be reversed when we know more about the consequences.^21^

This is a discussion that can be conducted in purely utilitarian terms. Even the human rights lawyers could redefine their position and say that international treaties offer such powerful protections that ignoring them would reduce happiness and welfare overall (they do not, and with good reason, but they could). It would be unwise, however, to limit the vocabulary to guesstimated consequences and their contested relative value. Both dimensions should alarm us. We do not have exhaustive knowledge about SARS-CoV-2, COVID-19, or the future development of the pandemic in different scenarios. The ends that we ought to pursue are equally unclear. Are survival and health supreme values, do human rights trump everything else, should we put economy first, or would it be good to reduce humankind and lighten the burden on other species and the natural environment?

## What Could Have Gone Right?

Finnish philosophers have not solved COVID-19-related problems by their public appearances. That was never expected. Even in the best case, their contribution is indirect and has to do with the preservation of the political system. Finland aspires to be a liberal democracy with strong commitments to the rule of law, transparent decisionmaking, and participatory governance. None of these is possible in the long run without public debates dissecting what governments do and on what grounds. In this sense, every polite opinion expressed in the media has been valuable.

Despite the doubts aired by Takala 15 years ago, timely firefighting could be just what bioethicists should do in crises like the COVID-19 pandemic. Well-formulated arguments could reveal the justifications of political choices and their background assumptions to citizens and decisionmakers alike. People could keep track of what the government is doing and why; and express their satisfaction or dissatisfaction in the next general election. Liberal democracy would flourish.^22^

Unfortunately, however, philosophical bioethics has never grown roots in Finland. Individuals have studied bioethical topics, but no master’s or doctoral programs exist. The two professors of medical ethics in the country teach healthcare personnel, but their contribution to the current discussion has been limited to a soothing interview^23^ and a short descriptive comment^24^ on prioritization.^25^ The human rights lawyers have their own competence, but in jurisprudence rather than in ethics. The other participants in the public exchange do not have track records in philosophical bioethics.

As a result, we have not seen conceptual analyses that would have carefully explicated, interpreted, and evaluated possible decisions and their grounds. Without experience in the field, it is all too easy to think that one’s own intuition is a sufficient guide, and to proceed directly to its rationalization by convenient arguments. At least my idea of philosophical bioethics is different, almost scientific, approaching a hypothetico-deductive scrutiny.^26^ This would explicate and interpret offered and other possible solutions by rational reconstruction, heeding to the principle of charity, and then evaluate the feasibility, acceptability, and effects of these solutions from different ethical viewpoints, including theories of justice.^27^ ^,^^28^ ^,^^29^ ^,^^30^

My model is not designed to produce the kind of direct, concrete impact that research funding organizations are currently looking for. Given time and resources, however, its implementation would make us better prepared for future crises and reasoned discussions on the political decisions involved.

## Notes

1. During March 26 to April 26, 2020, I followed, through my customary media and social media feed, the development of the COVID-19 pandemic, the actions that the government of Finland took to control it, and the public reactions of Finnish researchers whose work has to do with bioethics. I participated in the discussion on the blog site Etiikka.fi—and chronicled toward the end of my survey period my observations. Immediately after completing the survey, I sent an email inquiry to 35 bioethics-related academics in Finland and asked them to name recent ethical COVID-19 contributions. Very little of what they reported had escaped my attention, so either my coverage was reasonably representative, or all our feeds are similarly biased.

2.
https://fi.wikipedia.org/wiki/Koronaviruspandemia_Suomessa#Sulkutoimia_maalis-huhtikuussa (last accessed 26 Apr 2020).

3.
https://thl.fi/en/web/thlfi-en (last accessed 22 Apr 2020).

4.

Takala T. Demagogues, firefighters, and window dressers: What are we and what should we be? Cambridge Quarterly of Healthcare Ethics 2005;14:385–8.1625049210.1017/s096318010505053x

5. Häyry M. Päätöksenteon perustelu liberaalissa demokratiassa—Selittelyä mutta sille olisi paikkansa (Justifying decisions in liberal democracy—Rationalizations, but they have their place, in Finnish). *Etiikka.fi* 2020 Apr 6; available at https://etiikka.fi/paatoksenteon-perustelu-liberaalissa-demokratiassa-selittelya-mutta-sille-olisi-paikkansa/ (last accessed 26 Apr 2020).

6. Milstein C. Kollektiivinen hoiva on paras aseemme koronavirusta vastaan. *Takku* 2020 Mar 31; available at https://takku.net/article.php/2020033107451615#comments (last accessed 28 Apr 2020).

7. Godman M. Tänk om far dör ensam på demensboendet—Relationer på undantag i coronatider (What if father dies alone in a dementia home—Relations revoked in Corona times, in Swedish). *Hufvudstadsbladet* 2020 Apr 25.

8. As leading politicians argued that they are taking measures “for the overall benefit of the society,” already in mid-March philosopher Henrik Rydenfelt warned about the ambiguity of such concepts and the concentration on narrow measures at the expense of other aspects of well-being and basic rights. Rydenfelt H. Kenen on kokonaisetu? (Whose is the overall benefit?) Etiikka.fi 18 March 2020. Available online: https://etiikka.fi/kenen-on-kokonaisetu/ (last accessed 26 Apr 2020).

9. Scheinin M, Rautiainen P. Koronakriisin uusissa toimissa valmiuslain nojalla ollaan nyt poikkeamassa ihmisoikeussopimuksista—Valtioneuvosto leikkii jumalaa (In the new corona crisis decisions, the government employs the emergency powers law to stray from human right agreements and plays God, in Finnish). *Perustuslakiblogi* (*Constitutional Law Blog*) 2020 Mar 26; available at https://perustuslakiblogi.wordpress.com/2020/03/26/martin-scheinin-pauli-rautiainen-koronakriisin-uusissa-toimissa-valmiuslain-nojalla-ollaan-nyt-poikkeamassa-ihmisoikeussopimuksista-valtioneuvosto-leikkii-jumalaa/ (last accessed 23 Apr 2020).

10. Kauppinen A. Vahingon aiheuttaminen ei aina loukkaa oikeuksia (Causing harm does not always violate rights, in Finnish). *Etiikka.fi* 2020 Mar 27; available at https://etiikka.fi/vahingon-aiheuttaminen-ei-aina-loukkaa-oikeuksia/#comment-320 (last accessed 23 Apr 2020).

11. Kauppinen A, Kyllönen S. Perusoikeuksien rajoittaminen voi sopia poikkeusoloihin (Restrictions of basic rights can be acceptable during exceptional times, in Finnish). *Helsingin Sanomat* 2020 Apr 1; available at https://www.hs.fi/mielipide/art-2000006459283.html?share=0e62ea1da64e423a208729e3e0cc2cd8 (last accessed 23 Apr 2020).

12. Kauppinen A. Koronatoimien hinta ei ole sama asia kuin koronakriisin hinta (The price of corona action is not the same as the price of the corona crisis, in Finnish). *Suomen Kuvalehti* 2020 Apr 23; available at https://suomenkuvalehti.fi/jutut/kotimaa/filosofi-koronasta-vanhusten-viimeisia-elinvuosia-ei-saa-pyyhkia-pois-jotta-muille-maksimoitaisiin-laatupainotteista-elamaa/?fbclid=IwAR0O6Kt4L36E2oy_0v-wQXvpV2iShxCjaKXhgQWPsHnqhcIf83rzXhELrok (last accessed 24 Apr 2020).

13. Foot P. The problem of abortion and the doctrine of the double effect. *Oxford Review* 1967 May; available at http://www2.pitt.edu/~mthompso/readings/foot.pdf (last accessed 23 Apr 2020).

14. McIntyre A. Doctrine of double effect. *The Stanford Encyclopedia of Philosophy.* Spring 2019 ed. Zalta EN, ed; available at https://plato.stanford.edu/archives/spr2019/entries/double-effect/ (last accessed 23 Apr 2020).

15. Häyry M. Filosofialla voi todistaa mitä vain mutta myös lisätä päätöksenteon läpinäkyvyyttä (Philosophy proves anything but also increases transparency in decision making, in Finnish). *Etiikka.fi* 2020 Apr 1; available at https://etiikka.fi/filosofialla-voi-todistaa-mita-vain-mutta-myos-lisata-paatoksenteon-lapinakyvyytta/ (last accessed 23 Apr 2020).

16. See note 5, Häyry 2020.

17. See note 10, Kauppinen, Kyllönen 2020.

18. See note 14, Häyry 2020.

19. See note 10, Kauppinen, Kyllönen 2020.

20. Anonymous. THL:n arvio koronasta: Jos epidemian hidastuminen jatkuu pitkään, tulee suuri riski isoon epidemiaan myöhemmin (THL assessment on Corona: If the epidemic keeps slowing down for long, there is a sizeable risk of a big epidemic later, in Finnish); available at https://yle.fi/uutiset/3-11325177 (last accessed 27 Apr 2020).

21. See note 11, Kauppinen 2020.

22. See note 5, Häyry 2020.

23. Valtanen M. Tehohoito ei kuulu kaikille (Intensive care is not for everyone, in Finnish). *Suomen Kuvalehti* 2020 Apr 24; available at https://suomenkuvalehti.fi/jutut/kotimaa/tehohoito-ei-auta-kaikkia-koronapotilaita-siksi-on-valittava-ketka-hoidetaan-vaihtoehto-olisi-kaaos/?shared=1123563-fe0f54f3-999 (last accessed 26 Apr 2020).

24. Louhiala P. Kuka otetaan, kuka jätetään? (Who is chosen, who is not? in Finnish). *Suomen Lääkärilehti* 2020:952.

25. Häyry M. Priorisointiin ei ole hyvä joutua mutta pitäisikö siitä puhua? (Prioritization would be bad, but should we talk about it? in Finnish). *Etiikka.fi* 2020 Apr 8; available at: https://etiikka.fi/priorisointiin-ei-ole-hyva-joutua-mutta-pitaisiko-siita-puhua/ (last accessed 26 Apr 2020).

26.

Häyry M . What do you think of philosophical bioethics? Cambridge Quarterly of Healthcare Ethics 2015;24:139–48.2571934910.1017/S0963180114000449

27. Häyry M. Pandemian jälkeen—Tuhon tie vai järki käteen? (After the pandemic—The road to destruction or reasonable reassessment? in Finnish). *Etiikka.fi* 2020 Apr 12; available at https://etiikka.fi/pandemian-jalkeen-tuhon-tie-vai-jarki-kateen/ (last accessed 26 Apr 2020).

28. Häyry M. Tasapuolisten neuvonantajien tulevaisuus—Onko vielä toivoa? (The future of impartial advisers—Do we still have hope? in Finnish). *Etiikka.fi* 2020 Apr 14; available at https://etiikka.fi/tasapuolisten-neuvonantajien-tulevaisuus-onko-viela-toivoa/ (last accessed 26 Apr 2020).

29. Häyry M, Rydenfelt H. Vain parhaat täytteet—Tasapuolisten neuvonantajien calzone (Only the best fillings—The calzone of impartial advisers, in Finnish). *Etiikka.fi* 2020 Apr 20; available at https://etiikka.fi/vain-parhaat-taytteet-tasapuolisten-neuvonantajien-calzone/ (last accessed 26 Apr 2020).

30. Häyry M. Kopatia ja tasapuolisuus—Ilmenevätkö enkelit vain poikkeusoloissa? (Copathy and impartiality—Do angels appear only in exceptional circumstances? in Finnish). *Etiikka.fi* 2020 Apr 24; available at https://etiikka.fi/kopatia-ja-tasapuolisuus-ilmenevatko-enkelit-vain-poikkeusoloissa/ (last accessed 26 Apr 2020).

