# The COVID-19 Pandemic: A Month of Bioethics in Finland—ADDENDUM

**DOI:** 10.1017/S0963180120000511

**Published:** 2020-06-29

**Authors:** MATTI HÄYRY

**Keywords:** COVID-19, pandemic, bioethics, human rights, utilitarianism, doctrine of double effect.

## Abstract

The role of bioethicists amidst crises like the COVID-19 pandemic is not well defined. As professionals in the field, they should respond, but how? The observation of the early days of pandemic confinement in Finland showed that moral philosophers with limited experience in bioethics tended to apply their favorite theories to public decisions with varying results. Medical ethicists were more likely to lend support to the public authorities by soothing or descriptive accounts of the solutions assumed. These are approaches that Tuija Takala has called the firefighting and window dressing models of bioethics. Human rights lawyers drew attention to the flaws of the government’s regulative thinking. Critical bioethicists offered analyses of the arguments presented and the moral and political theories that could be used as the basis of good and acceptable decisions.

Owing to an editorial oversight, the abstract and keywords were omitted from the original online version of the article by Häyry (published online 30 April 2020).^1^ The abstract and keywords are as follows:

The abstract and keywords have been added to the online version of the article.

## Note

1. Häyry M. The COVID-19 pandemic: A month of bioethics in Finland. *Cambridge Quarterly of Healthcare Ethics.* Published online 30 April 2020. https://doi.org/10.1017/S0963180120000432.

